# A144 BLACK ESOPHAGUS: A PEDIATRIC PRESENTATION OF ACUTE ESOPHAGEAL NECROSIS AND MANAGEMENT OF ITS COMPLICATIONS

**DOI:** 10.1093/jcag/gwac036.144

**Published:** 2023-03-07

**Authors:** N Almansour, A Balbaa, R Sharma, J Dowhanuik, M Sherlock, M Zachos, H Brill

## Abstract

**Background:**

Acute esophageal necrosis (AEN), or "Black Esophagus", is a rare diagnosis in children with adult literature citing high mortality. We present a pediatric case of this unusual condition.

**Purpose:**

To report the rare presentation of AEN in a child and the response to intensive endoscopic management.

**Method:**

Chart and literature review of AEN presentation and management.

**Result(s):**

A 13-year-old male who presented with a one-day history of severe chest pain, worsened with swallowing, followed by coffee ground emesis. No caustic ingestion. A nasogastric (NG) tube was placed and 2L of coffee-ground aspirate drained.

The patient was an ex-preterm infant (25 weeks) who presented at age 2 years with an antral ulcer that developed into an antral stricture. This resolved with successive endoscopic dilatation with no recurrence after 5 years, off acid suppression.

Physical exam showed BMI 99th %ile and palpated epigastric tenderness.

CT-angiogram showed diffuse circumferential thickening of the esophagus and impressive gastric dilatation. A contrast study showed stasis of contrast in the esophageal lumen.

Upper endoscopy revealed a thick, white membrane followed by circumferential black mucosa in the mid to distal esophagus. Previous antral stricture was noted with easy scope passage. Biopsies were negative for fungus, bacteria, HSV, CMV and parvovirus. Inflammatory infiltrate (mainly neutrophils and scattered histocytes with occasional lymphocytes) was noted in the esophagus. Necrotic tissue was identified and a diagnosis of AEN was made.

The patient remained NPO, on TPN, and morphine was used for pain control. He responded well to this, along with high dose PPI. He tolerated clear fluids and oral sucralfate. Repeat scope one week later showed resolved necrosis with remaining oedematous white plaques and an unchanged antral stricture.

10 days later he developed severe dysphagia and a 15.5 cm long 6 mm diameter distal esophageal stricture was identified. This was managed by serial balloon dilatations. Due to frequent need for dilatations, endoscopic triamcinolone injections were used in several procedures as an adjunct therapy. A total of 80 mg of triamcinolone per session was injected along the length of the stricture in 4 aliquots in five out of nine endoscopic dilations in the last seven months. The most recent procedure successfully dilated the esophagus to 18mm.

Currently, the patient is now tolerating a soft oral diet and remains on PPI therapy.

**Image:**

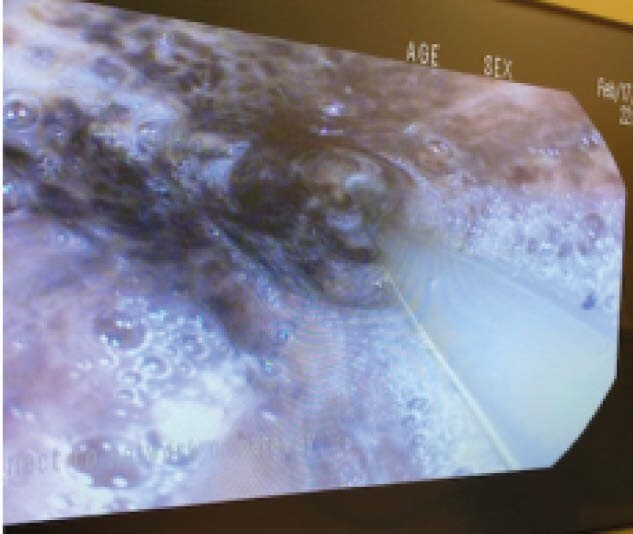

**Conclusion(s):**

AEN is very rare in children with no literature describing the endoscopic management of AEN related stenotic complications. We report the case of this unique presentation in a teenager, resulting in severe, long-segment esophageal stenosis. Intensive endoscopic therapeutic management has resulted in a positive clinical outcome allowing oral nutrition without any stenting or surgical interventions. Early recognition and active endoscopic management likely contributed to the positive outcome.

**Please acknowledge all funding agencies by checking the applicable boxes below:**

None

**Disclosure of Interest:**

None Declared

